# Tankyrase 1 and Tankyrase 2 Are Essential but Redundant for Mouse Embryonic Development

**DOI:** 10.1371/journal.pone.0002639

**Published:** 2008-07-09

**Authors:** Y. Jeffrey Chiang, Susan J. Hsiao, Dena Yver, Samuel W. Cushman, Lino Tessarollo, Susan Smith, Richard J. Hodes

**Affiliations:** 1 Experimental Immunology Branch, National Cancer Institute, National Institutes of Health, Bethesda, Maryland, United States of America; 2 Center for Cancer Research, Mouse Cancer Genetics Program, National Cancer Institute, Frederick, Maryland, United States of America; 3 The Kimmel Center for Biology and Medicine of the Skirball Institute, New York University School of Medicine, New York, New York, United States of America; 4 National Institute of Diabetes and Digestive and Kidney Diseases, National Institutes of Health, Bethesda, Maryland, United States of America; 5 National Institute on Aging, National Institutes of Health, Bethesda, Maryland, United States of America; Duke University, United States of America

## Abstract

Tankyrases are proteins with poly(ADP-ribose) polymerase activity. Human tankyrases post-translationally modify multiple proteins involved in processes including maintenance of telomere length, sister telomere association, and trafficking of glut4-containing vesicles. To date, however, little is known about in vivo functions for tankyrases. We recently reported that body size was significantly reduced in mice deficient for tankyrase 2, but that these mice otherwise appeared developmentally normal. In the present study, we report generation of tankyrase 1-deficient and tankyrase 1 and 2 double-deficient mice, and use of these mutant strains to systematically assess candidate functions of tankyrase 1 and tankyrase 2 *in vivo*. No defects were observed in development, telomere length maintenance, or cell cycle regulation in tankyrase 1 or tankyrase 2 knockout mice. In contrast to viability and normal development of mice singly deficient in either tankyrase, deficiency in both tankyrase 1 and tankyrase 2 results in embryonic lethality by day 10, indicating that there is substantial redundancy between tankyrase 1 and tankyrase 2, but that tankyrase function is essential for embryonic development.

## Introduction

The tankyrase protein family consists of tankyrase 1 and 2, which share 85% amino acid sequence identity [1], [2]. Tankyrase 1 contains 24 ankyrin (ANK) repeats near the N-terminus, that mediate interaction with multiple proteins, including TRF1 (telomere-repeat binding factor-1) [2] , IRAP (insulin-responsive aminopeptidase) [3], TAB182 (182-kDa tankyrase-binding protein) [3], [4], and NuMA (nuclear/mitotic apparatus protein) [3], [5]. The C-terminus of tankyrase contains a catalytic domain with poly(ADP-ribose) polymerase (PARP) activity [2]. A Sterile Alpha Module (SAM), another protein binding motif, lies between these terminal domains [2].

Tankyrase proteins have been reported to be present in both the nucleus and cytoplasm, and recent publications suggest that tankyrase 1 might play multiple functions through various interacting proteins, reviewed in [6]. The role of human tankyrase has been extensively assessed in maintenance of telomeres. Telomeres are structures at the end of eukaryotic chromosomes, consisting of hexanucleotide repeats and associated proteins, which play a critical role in cellular senescence, apoptosis and tumorigenesis, reviewed in [7], [8]. Telomere length is regulated by the enzyme telomerase, which consists of a catalytic telomerase reverse transcriptase (TERT) and an RNA template (TR) that encodes the telomeric DNA sequence, reviewed in [9]. Tankyrase 1 has been shown to co-localize with TRF1 to telomeres [2]. Overexpression of human tankyrase 1 results in release of TRF1 from telomeres and telomere elongation in human cell lines, whereas overexpression of mutated tankyrase 1 lacking PARP activity fails to affect TRF1 and telomere length [10], [11]. These studies have lead to the hypothesis that tankyrase 1 ADP-ribosylates TRF1, releasing it from telomeric complexes, resulting in a telomeric conformation that provides greater access of telomerase to telomeres.

SiRNA-mediated knockdown of human tankyrase 1 has been shown to result in cell cycle arrest at mitosis [12], [13], with an increase in persistent sister telomere association [13] and spindle dysfunction [12]. The amount of tankyrase 1 and the intensity of poly(ADP-ribose) (PAR) staining of the spindle are correlated in tankyrase1 siRNA knockdown cells [12], suggesting that the PARP activity of tankyrase 1 might promote bipolar spindle assembly by ADP-ribosylating spindle components such as NuMA, an acceptor of ADP-ribosylation by tankyrase in mitosis [5], [12]. Recently, it was shown that the requirement for tankyrase 1 in sister telomere resolution and mitotic progression could be abrogated by depletion of TRF1 and its binding partner TIN2 or the cohesin subunit SA1, suggesting that unresolved cohesion at telomeres might promote persistent sister telomere association and mitotic arrest [14].

In addition to telomere-related functions, it has been reported that knockdown of human tankyrase 1 in adipocyctes inhibits translocation of the glucose transporter Glut4 and insulin-regulated aminopeptidase (IRAP) from endosomal compartments to the plasma membrane, altering intracellular Glut4/IRAP distribution [15]. Glut4 and IRAP are among the cargo of Glut4 storing vesicles (GSV), and it was suggested that GSV trafficking depends upon the presence of IRAP in these vesicles, which in turn is regulated by tankyrase via its PARP activity [15], [16]. It has therefore been proposed that tankyrases may regulate insulin-dependent glucose metabolism through their effect on GSV.

Although tankyrase 2 has been less extensively studied than tankyrase 1, the structural homology and protein binding properties of tankyrase 1 and 2 suggest that tankyrase 2 might have similar or redundant functions with tankyrase 1. The function of human tankyrase 2 in telomere length maintenance has been shown to be similar to that of tankyrase 1. Overexpression of wild type tankyrase 2 in human cells resulted in telomere elongation, while overexpression of tankyrase 2 mutated in the PARP domain failed to induce elongation [6]. Whether human tankyrase 2 mediates functions similar to those described for tankyrase 1 in separation of sister telomeres and mitotic progression remains to be determined. It is similarly unclear whether tankyrase 2 functions in GSV transport and glucose uptake in a manner similar to tankyrase 1. It was reported that tankyrase 2 can interact with IRAP in a yeast two hybrid system [17]; however, the involvement of tankyrase 2 in regulation of GSV trafficking has not been defined.

Recently, we reported *in vivo* studies of tankyrase 2 function using mice genetically engineered to be completely deficient in TANK2 or to express a PARP-deficient TANK2 mutation. TANK2 deficient mice developed normally and exhibited no detectable change in telomere length, but did show a significant decreases in total body weight that might reflect a role of tankyrase 2 in glucose/fat metabolism [6], [18]. The absence of other changes, including those in telomere length, could reflect a redundancy of tankyrase 1 and tankyrase 2 functions [6], [18]. Therefore, to further understand the *in vivo* functions of tankyrases, we have generated TANK1 knockout mice and TANK1/2 double deficient mice. In the present study, we report a systematic analysis of putative tankyrase functions in mice deficient in either tankyrase 1 or tankyrase 2, as well as the phenotype resulting from deficiency in both tankyrase 1 and tankyrase 2.

## Materials and Methods

### Targeting vector, electroporation, and selection

A tankyrase 1 gene targeting vector was constructed from an 11 kb SalI/KpnI fragment of mouse genomic DNA containing the first exon of tankyrase 1. A loxP sequence was inserted upstream of exon 1, and a loxP flanked neo gene was inserted in intron 1 in a direction opposite that of the tankyrase 1 gene to serve as a positive selectable marker. The thymidine kinase cassette (TK) was put further upstream of exon 1 and used as a negative selectable marker. Electroporation and selection were performed with the CJ7 embryonic stem (ES) cell line as described by Tessarollo [19].

### Generation of tankyrase 1 deficient mice and tankyrase 1/2 double deficient mice

Two independent TANK1 targeted ES cell clones were injected into C57BL/6 (B6) blastocysts, generating chimeras that transmitted the targeted allele to progeny. The heterozygous offspring were bred to generate homozygous mice that were named as conditional TANK1 knockout mice or floxed mice (TANK1^flox/flox^). The floxed mice were bred with EIIa-cre mice [20] that express the loxP-specific DNA recombinase, cre, at embryonic day 1 and 2. Mice that had deleted TANK1 exon 1 at one allele were selected from the offspring of TANK1^flox/flox^ X EIIa-cre crosses. Mice homozygous for the deletion of TANK1 exon 1 were designated as constitutive TANK1 knockout mice (TANK1^−/−^). TANK1^−/−^ mice were bred with TANK2^−/−^ mice [18] to generate TANK1^−/−^TANK2^−/−^ double knockout mice. All animals were housed at Bioqual (Rockville, MD).

### Southern blot, PCR analysis for genotype

**Figure 1:**
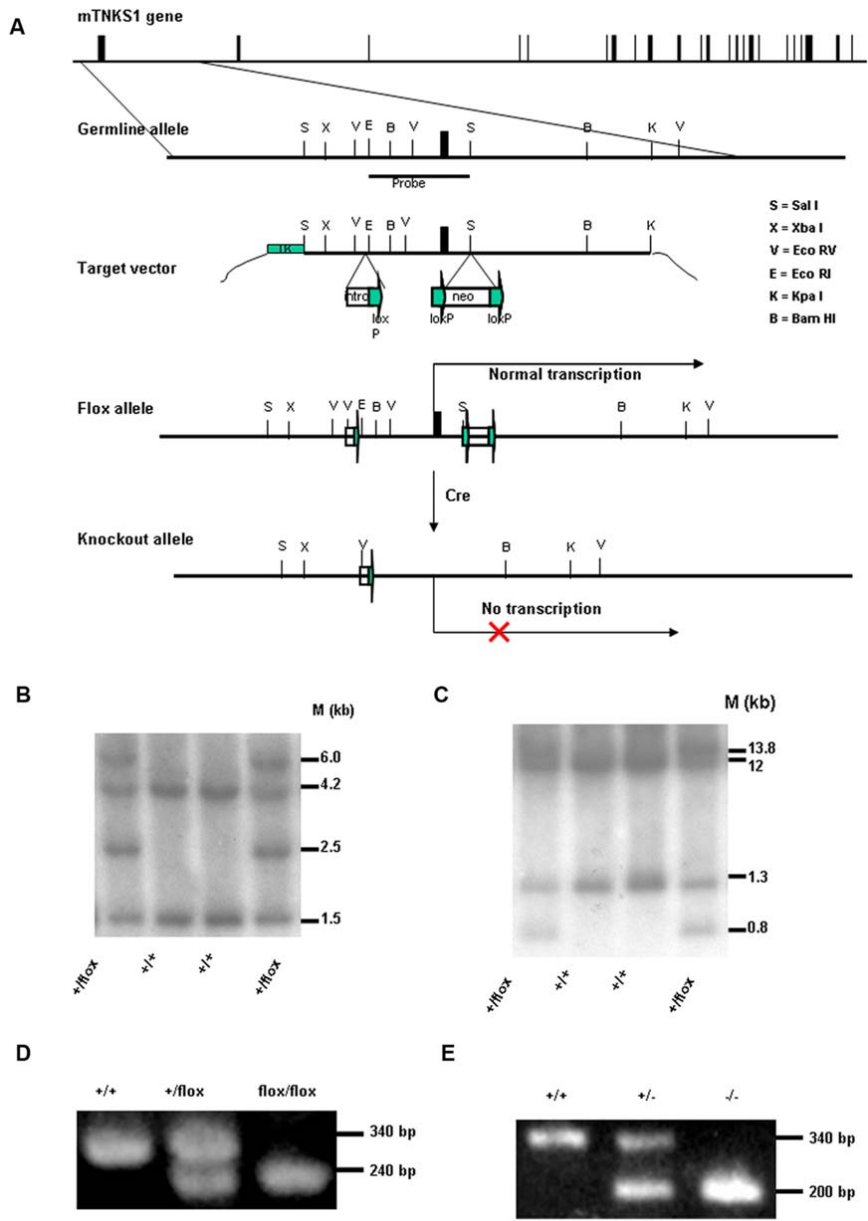
Generation of TANK1-deficient mice by gene targeting. (A) Gene targeting strategy and restriction map of TANK1 gene. Filled boxes indicate exons; labeled boxes indicate neomycin (neo) resistance or herpesvirus thymidine kinase (tk) genes; and arrows indicate loxP sites. (B, C) Southern blot analysis of ES cell DNA. The 4.2 kb and 1.5 kb bands represents the germ line alleles and 6.0 kb and 2.5 kb bands represent the targeted alleles when BamHI/XbaI enzymes were used (B). The 12 kb and 1.3 kb bands represent the germ line alleles, and 13.8 kb and 0.8 kb bands represent the targeted alleles when EcoRV was used(c). (D) PCR analysis for the conditional TANK1 knockout mouse genotype. The 340- and 240-bp PCR products represent the wild-type and floxed alleles, respectively. (E) PCR analysis for the constitutive TANK1 knockout mouse genotype. The 340- and 200-bp PCR products represented the wild-type and knockout alleles, respectively.

DNA for Southern blot analysis was isolated from ES cells and mouse tails. DNA isolation and Southern blot analysis procedures have been described elsewhere [18]. DNA was digested with BamHI/XbaI or EcoRV, electrophoresed on a 0.7% agarose gel, transferred to a nylon membrane, and probed as indicated in Figure 1a. Tail DNAs were prepared with the automatic DNA isolation system of Autogen (Framingham, MA) for PCR genotyping. PCR primers were TNK1S2F (5′-TTT TCA GTT CAG AGT GTG CC-3′), TNK1S2R (5′-GTC TCT CTC TGC CCC TTA TC-3′), LOXP (5′-TGG CTG GAC GTA AAC TCC TCT TCA GAC CTA ATA AC-3′), NEO1 (5′-TTC TGG ATT CAT CGA CTG TG-3′). PCR reactions were carried out in 50 µl of PCR reaction mix containing 25 µl of PCR buffer (Supermix PCR kit, Life Technologies, Rockville, MD), 1–100 ng of tail DNA, and in the presence of either the NEO1 primer set (0.2 µM of each TNK1S2F, TNK1S1R and NEO1 primer) or the LOXP primer set (0.2 µM of each TNK1S2F, TNK1S1R and LOXP primer). PCR was carried out at 95°C for 30 sec, 58°C for 30 sec and 72°C for 1 min. PCR products were revealed by agarose gel electrophoresis and ethidium bromide staining. The 340-bp band corresponds to the wild-type allele, the 240-bp band corresponds to conditional knockout allele, and the 200-bp band corresponds to both conditional and constitutive knockout allele.

### Reverse transcription PCR

Various tissues from wild-type and TANK1−/− mice were used to isolate RNA with a NucleoSpin kit (BD Biosciences). Total RNA was used for RT-PCR analysis. Three primer pairs were used to detect TANK1 expression: for detection of 5′-, 3′- tankyrase 1 and tankyrase 1a cDNA. RT-PCR reactions were carried out in 50 µl of PCR reaction mix containing 25 µl of PCR buffer (One-step RT-PCR kit, Life Technologies), 1 µg of total RNA and 0.2 µM primers. RT-PCR was carried out at 42°C for 30 minutes, 35 cycles of 95°C for 30 sec, 58°C for 30 sec and 72°C for 1 min. RT-PCR products were visualized by agarose gel electrophoresis with EtBr staining. 5′-tankyrase 1 cDNA PCR product (337-bp) was visualized when the primer pair (TNK1C632F: 5′-AGT CTT CTC CCC TGC ACT TC-3′ and TNK1C969R: 5′-AGC TTT TGC TGA AGG ATC TG-3′) was used. 3′-tankyrase 1 cDNA PCR product (299-bp) was visualized when the primer pair (TNK1C1175F: 5′-CGA AAG ACA AAG GTG GAC TTG-3′ and TNK1C1474R: 5′-CGG CTT GGA GTA AAG AAT GG-3′) was used. Tankyrase 1a RT-PCR product (425-bp) was visualized when the primer pair (TNK1AF151: 5′-GAT CAT TTC TAA GTG AAG TT-3′ and TNK1AR576: 5′-GTC CAC CTT TGT CTT TCG C-3′) was used.

### Western blot analysis

Twenty-five micrograms of protein extracts, prepared from various tissues of wt and TANK1^−/−^ mice, were fractionated by sodium dodecyl sulfate-polyacrylamide gel electrophoresis and transferred to a nitrocellulose membrane. Immunoblot analysis was carried with rabbit anti-tankyrase 1 antibodies 376 [10] and 762 [21].

### Flow-FISH

Spleen lymphocytes were prepared and incubated with FITC-labeled (CCCTAA)_3_ oligonucleotide in buffer containing 20 mM TRIS, 75% formamide, 1% BSA and 20 mM NaCl at 86°C for 15 min, followed by room temperature for 2 hours in the same buffer [22], [23]. Cells were used for FACS analysis after washing 4 times with buffer containing 20 mM TRIS, 75% formamide, 1% BSA and 0.1% Tween 20.

### Cell cycle analysis

Spleen cells were prepared and stimulated with Con A (5 µg/ml), LPS (15 µg/ml), and rIL2 150 IU/ml) for 48 hours with or without 10 Gy gamma irradiation after the initial 24 hours of culture. Following culture, cells were fixed with 70% ethanol overnight at −20°C. The fixed cells were stained with 10 µg/ml of PI and 1 µg/ml RNAase for 30 minutes and analyzed by FACS [13].

### In vitro assay for glucose uptakes in mouse WAT cells

The preparation of white adipose tissue cells and measurement of glucose uptake were as previously described [24]. Briefly, the epididymal fat pads were removed, minced, and digested with collagenase (10.5 mg/ml) in KRBH buffer (Krebs-Ringer-bicarbonate-HEPES buffer, pH = 7.4) at 37°C for 2 hours. The adipocytes were obtained by further washing with KRBH buffer. The adipocyte suspension was added to 150 µl KRBH, 5% albumin buffer in the absence or presence of insulin (0.5 nM), and incubated at 37°C for 30 minutes after addition of 3-O-[methyl-1-14C]methylglucose (0.083 µCi) (New England Nuclear; Boston,MA) in 100 µl KRBH buffer. 200 µl of cell suspension was mixed with 100 µl of dinonylphthalate, and centrifuged for 1 min. The radioactivity (cpm) of upper portion, which contained packed cells, was determined by Packard Tri-Carb scintillation counter.

### Embryo isolation and histology

Timed-pregnant mice were made by intercrossing TANK1+/−TANK2+/− double heterozygous mice, and embryos were isolated at specific times of embryonic development. Embryos were fixed in 4% paraformaldehyde at 4°C overnight, dehydrated through a graded alcohol series, and then embedded in paraffin. Sections of 6–8 µm were prepared and stained with standard H/E protocols [25].

## Results

We recently reported that mice that are completely deficient in TANK2 expression [18], or mice that express only a mutated form of TANK2 lacking PARP activity [6], manifest similar phenotypes with limited differences from wild type controls. TANK2 knockout and mutant mice exhibited a significant reduction in body weight, but otherwise grossly normal development [6], [18]. Human tankyrases have been shown to be critical factors for telomere length maintenance. However, no abnormality in telomere length was observed in early generation TANK2-deficient mice, suggesting that tankyrase 1 and 2 might have redundant functions in telomere length maintenance [6], [18]. Here, we generated tankyrase 1 knockout and tankyrase 1 and 2 double knockout mice to assess the physiological roles of tankyrases.

### Generation and Characterization of TANK1 Knockout mice

Cre/loxP technology was used to generate a tankyrase 1 conditional knockout mouse that was converted into a constitutive knockout by crossing with EII-cre transgenic mice, similar to the previously reported strategy for generating tankyrase 2 deficient mice [18]. Figure 1A illustrates the strategy for tankyrase 1 conditional and constitutive gene targeting. TANK1 conditional knockout ES clones were identified by Southern blot analysis. For Southern analysis, ES cell genomic DNA was digested with BamHI/XbaI or EcoRV, electrophoresed, and hybridized with probes (Fig. 1A). Figure 1B shows the results of a representative Southern blot in which wild-type ES cell DNA generated 4.2 and 1.5 kb bands with probe when BamHI/XbaI was used, while heterozygous conditional knockout ES cell DNA generated two additional 6.0 and 2.5 kb bands with probe. Wild-type ES cell DNA generated 1.3 and 12 kb bands with probe when EcoRV was used, while heterozygous conditional knockout ES cell DNA generated two additional 0.8 and 13.8 kb bands (Fig. 1C). Four heterozygous ES cell clones were identified from screening of 137 clones, and two of these clones were used to generate conditional TANK1 knockout mice. Mouse genotypes were identified using genomic PCR with primers TNK1S2F, NEO1 and TNK1S2R. Figure 1D shows that DNA from wild-type mice (TANK1^+/+^) gave one band of 340 bp, DNA from homozygous floxed mice (TANK1^flox/flox^) one band of about 240 bp, and DNA from heterozygous mice (TANK2^+/flox^) bands of both 340 bp and 240 bp.

To determine the effect of TANK1 inactivation *in vivo*, we next generated constitutive TANK1 knockouts from TANK1^flox/flox^ parental mice. Conditional TANK1 knockout mice (TANK1^flox/flox^) were bred with EIIa-cre transgenic mice that express cre recombinase at embryonic day 1 and 2. The resulting knockout mice were identified by genomic DNA PCR with primers TNK1S2F, LOXP and TNK1S2R. As shown in Figure 1E, wild-type mice (TANK1^+/+^) gave one band (around 340 bp), homozygous knockout mice (TANK1^−/−^) one band (around 200-bp), and heterozygous mice (TANK1^+/−^) both 340-bp and 200-bp bands. Thirty-four TANK1^+/+^, sixty-six TANK1^+/−^, and thirty-five TANK1^−/−^ mice were observed in 135 offspring from TANK1^+/−^ intercrosses, consistent with Mendelian segregation and indicating that no lethality was associated with complete TANK1 inactivation.

**Figure 2:**
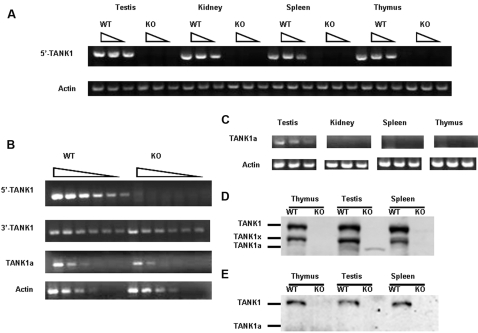
Expression of TANK1 and TANK1a in WT and TANK1^−/−^ mouse tissues. All RNA samples were serially diluted as indicated for PCR amplification and analysis. Actin cDNA was used as an RT-PCR loading control in all experiments. (A) RT-PCR analysis for TANK1 mRNA expression in various tissues of WT and TANK1^−/−^ (KO) mice as indicated. 5′-TANK1 RT-PCR products represent upstream cDNA of TANK1. (B) RT-PCR analysis for TANK1 mRNA expression in WT and TANK1^−/−^ (KO) testis as indicated. 5′-TANK1 and 3′-TANK1 RT-PCR products represent upstream and downstream cDNAs of TANK1; and TANK1a RT-PCR products are specific for TANK1a. (C) RT-PCR analysis for TANK1a mRNA expression in various tissues of WT mice as indicated. TANK1a RT-PCR products represent cDNA of TANK1a. (D, E) Western blot analysis was used to determine tankyrase 1 and 1a expression in thymus, testis and spleen of WT and TANK1^−/−^ (KO) mice with 762 (anti-SAM, D) and 376 (anti-HPS, E) antibodies. TANK1 indicates tankyrase 1 protein, TANK1x indicates a possible degraded tankyrase 1 protein, and TANK1a indicates tankyrase 1a protein.

Expression of tankyrase 1 was determined by RT-PCR with primers TNK1C632F/ TNK1C969R set and TNK1C1175F/TNK1C1474R set as described above. The 5′-TANK1 cDNA (337 bp) PCR products were detected in testis, kidney, spleen and thymus of wild-type mice while no 5′-TANK1 cDNA from RT-PCR product was detected in various tissues of homozygous (TANK1^−/−^) knockout mice (Fig. 2A). To assess presence of tankyrase protein, lysates from kidney, thymus, testis and spleen of WT and TANK1^−/−^ mice were immunoblotted with anti-tankyrase 1 antibodies. Figure 2D shows the results of immunoblotting lysates of WT and TANK1^−/−^ thymus, testis and spleen with anti-tankyrase 1 (762) antibody against the tankyrase 1 SAM domain. No tankyrase 1 protein was detected in TANK1^−/−^ thymus, testis or spleen while tankyrase 1 was found in all WT tissues (Fig. 2D). An additional lower molecular weight band, designated TANK1x, was also present in lysates from all WT, but not TANK1^−/−^ tissues tested, and is a possible TANK1 degradation product. Immunoblotting was also performed with antibody (376) specific for tankyrase 1 HPS domain determinants. This antibody also detected tankyrase 1 protein in tissues from WT but not TANK1−/− mice (Fig. 2E). RT-PCR and immunoblotting data thus indicate that transcription of the tankyrase 1 gene had been disrupted in this mouse model. Unexpectedly, a small protein band, designated as TANK1a in Figure 2D, was detected in both WT and TANK1^−/−^ testis, as discussed further below.

### Altered tankyrase 1 gene expression in TANK1−/− mice

**Figure 3:**
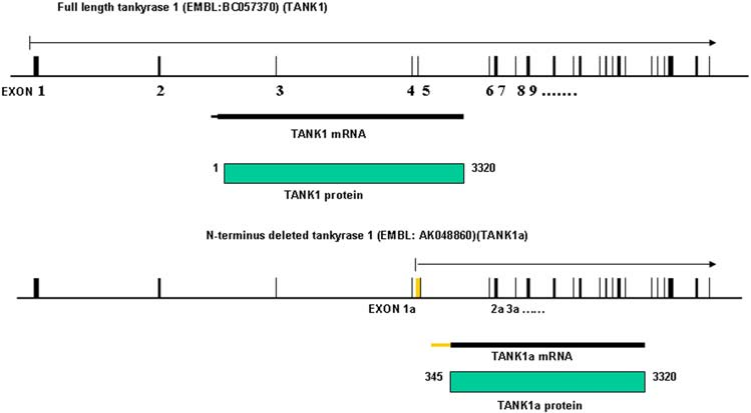
Genomic structure, mRNA and protein product for TANK1 and TANK1a.

RT-PCR analysis of TANK1 expression using primers specific for either the 3′ or 5′ region of the TANK1 gene product yielded unexpected findings. 3′-TANK1 RT-PCR product was detected at similar levels in WT and TANK1^−/−^ testis, while 5′-TANK1 RT-PCR product was detected only in WT testis, and was completely absent in TANK^−/−^ testis (Fig. 2B). Analysis of the Genebank database sequence indicates that TANK1 has a potential alternative promoter capable of producing an alternative transcript which corresponds to a previously reported EST sequence (Accession #AK046840) and which could be translated into a HPS (region containing homopolymeric runs of His, Pro, and Ser) domain-deleted tankyrase 1 protein, which we designate as TANK1a (Fig. 3). A lower molecular weight protein species was detected in lysates from WT and TANK^−/−^ testis, but not in other tissues, by immunoblotting with an anti-tankyrase 1 antibody specific for the tankyrase 1 SAM domain (Fig 2D). This could correspond to the protein of TANK1a, because the tankyrase 1 knockout was generated in the present study by removal of the (first) promoter region and exon 1 of the TANK gene. The alternative promoter of the gene remains intact in the TANK1^−/−^ mouse and is potentially functional. The lower molecular weight TANK1a band was detected in both WT and TANK1−/− testis by immunoblotting with antibody 763 (made against full length tank1, data not shown), antibody 762 (anti-SAM) (Fig. 2D) and antibody 465 (anti-SAM, data not shown), but not with antibody 376 (anti-HPS, Fig. 2E). To understand the transcriptional expression of tankyrase 1a, the TANK1a expression-specific primers (TNK1AF151 and TNK1AR576 set) were used for RT-PCR analysis. The TANK1a-specific RT-PCR showed that WT and TANK1^−/−^ testis expressed similar levels of TANK1a (Fig. 2B). TANK1a-specific RT-PCR was also used to test the pattern of tissue-specific expression. RT-PCR product of TANK1a was detected only in WT testis, and not in kidney, spleen, or thymus (Fig. 2C). The RNA expression pattern of TANK1a thus correlated with expression of the protein tankyrase 1a (Fig. 2D). The low molecular weight band in Figure 2D is therefore likely to represent the TANK1a protein, and the TANK1 knockout model generated here appears to have effectively inactivated expression of the previously defined TANK1 protein, but to allow expression of TANK1a, a previously unrecognized alternatively expressed TANK1 product.

### Telomere length was not altered in TANK1 knockout mice

**Figure 4:**
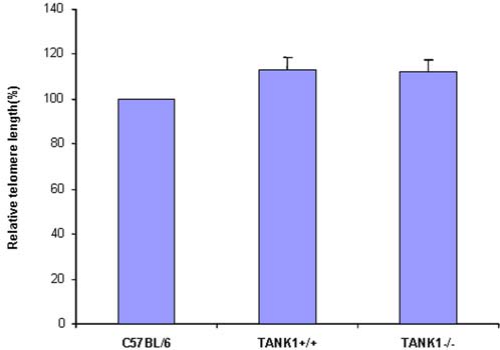
Telomere length was not altered in TANK1 knockout mice. Spleen cells were isolated from C57BL/6, TANK1^+/+^ (n = 7) and TANK1^−/−^ (n = 7) mice, and relative telomere length was determined by Flow-FISH. The FITC fluorescent signal of the cell-binding telomeric probe was converted to arbitrary units of molecule equivalents of soluble fluorescence (MSEF). The average of fluorescent intensities from each mouse was normalized to that of a C57BL/6 mouse (defined as 100). The relative telomere length of each strain of mice is plotted.

Previous *in vitro* studies have indicated that tankyrases influence telomere length in a telomerase-dependent manner [10], [26], and that interfering with tankyrase function can lead to decreasing telomere length, possibly through decreased access to telomerase (11, 24). However, we recently reported that inactivation of TANK2 in TANK2^−/−^ mice failed to show any effect on *in vivo* telomere length maintenance. It remained possible that the tankyrase family member tankyrase 1 might play a role in telomere maintenance or that tankyrase family members tankyrase 1 and tankyrase 2 might play significant but redundant roles in telomere length maintenance. To first determine whether tankyrase 1 plays an essential role in telomere length maintenance, spleen cells from seven TANK1^−/−^ knockout mice and the same number of their wild-type littermates were analyzed for telomere length by Flow-FISH. This analysis demonstrated that there was no detectable telomere shortening in TANK1^−/−^ knockout mice in comparison to littermate controls (TANK1^+/+^) (Fig. 4). No abnormal chromosome fusion events were observed in TANK1^−/−^ mice, consistent with absence of telomere dysfunction (data not shown). Thus, inactivation of TANK1^−/−^ had no effect on telomeres in first generation deficient mice. There was also no detectable telomere shortening in second to fifth generations of TANK1^−/−^ mice (data not shown). In contrast, telomere shortening was detected in first generation mTR or mTERT null mice [27], [28], as well as in mTR^+/−^ or mTERT^+/−^ heterozygotes [29], [30].

### No mitotic arrest was observed in TANK1^−/−^ or TANK2^−/^− mice

**Figure 5:**
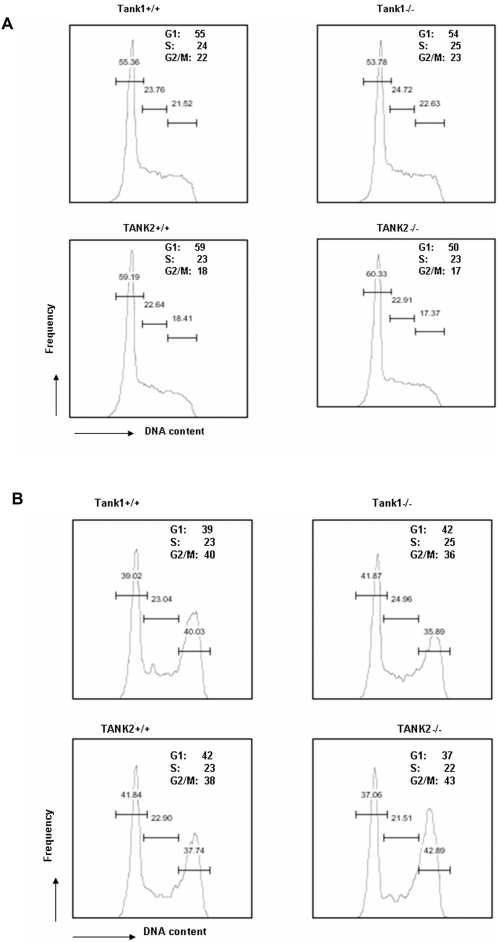
Cell cycle progression is equivalent in wild-type, TANK1^−/−^, and TANK2^−/−^ mice. (A) Spleen cells from TANK1^−/−^, TANK2^−/−^ mice and their littermates were stimulated with IL-2, ConA and LPS for 48 hours, then fixed and stained with PI. The results shown are representative of 3 independent analyses. The percentages of G1, S and G2/M phase are indicated. (B) Spleen cells from TANK1^−/−^, TANK2^−/−^ mice and their littermates were stimulated with IL-2, ConA and LPS for 48 hours, gamma irradiated at the 24 hour time point, then fixed and stained with PI. The results shown are representative of 3 independent analyses. The percentages of G1, S and G2/M phase are indicated.

HeLa cells transfected with human tankyrase 1 siRNA exhibited persistent sister telomere association and cell cycle arrest at G2/M [13]. To understand the involvement of mouse tankyrase proteins in cell cycle progression, cell cycle analysis was performed in stimulated splenocytes of TANK1^−/−^ and TANK2^−/−^ mice. As shown in Figure 5, there was no cell cycle arrest in either TANK1^−/−^ or TANK2^−/−^ mitogen-stimulated lymphocytes in comparison to wild-type cells (Fig. 5A), while normal and equivalent G2/M phase arrest was observed in X-ray irradiated WT, TANK1^−/−^ and TANK2^−/−^ lymphocytes (Fig. 5B). There thus appears to be no defect in cell cycling or irradiation-induced cell cycle response in TANK1^−/−^ or TANK2^−/−^ cells.

### Normal body weight observed in TANK1^−/−^ mice

**Figure 6:**
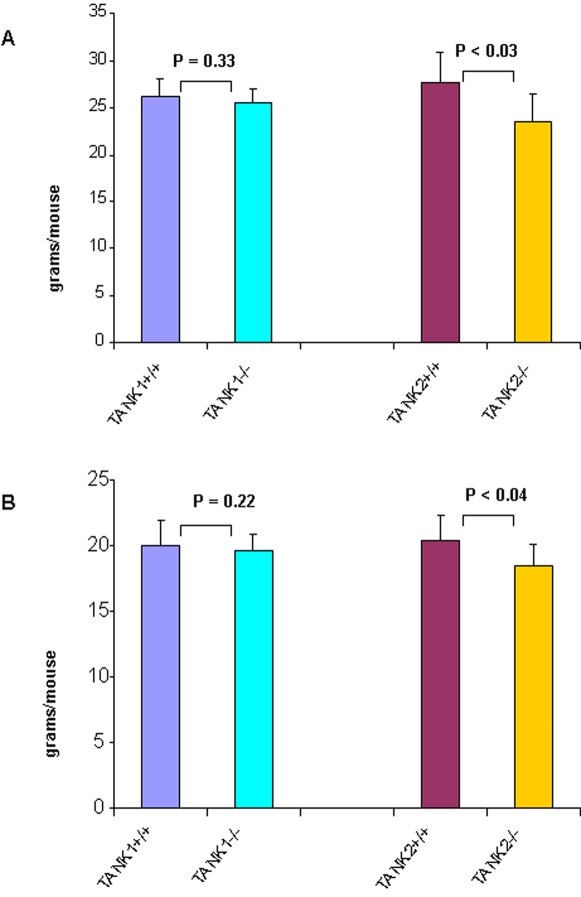
The body weights of tankyrase 1 knockout mice are normal while the body weights of tankyrase 2 knockout mice are reduced. (A) Body weights in male TANK1^+/+^ (n = 8), TANK1^−/−^ (n = 8), TANK2+/+ (n = 5) and TANK2−/− (n = 5) mice. (B) Body weights in female TANK1^+/+^ (n = 5), TANK1^−/−^ (n = 5), TANK2^+/+^ (n = 4) and TANK2^−/−^ (n = 4) mice.

We previously found that the body weight of TANK2−/− mice is significantly decreased relative to wild type controls. To determine whether tankyrase 1 might mediate similar functions in mouse growth, the body weight of 8 to 9 week-old WT and TANK1^−/−^ mice was measured. No significant difference in body weight was found between male WT and TANK1^−/−^ mice (Fig. 6A) or in the comparison between females (Fig. 6B), in contrast to the finding that body weights of male or female TANK2^−/−^ mice were significantly less than that of male or female WT mice, respectively (Fig. 6A, B).

### Insulin-stimulated glucose uptake in TANK2^−/−^ and TANK1^−/−^WAT cells

**Figure 7:**
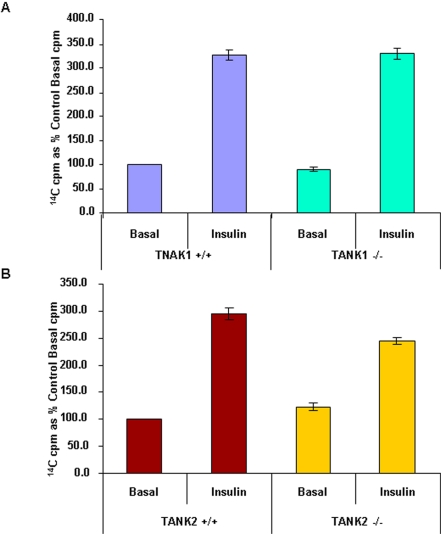
The sensitivity of insulin-stimulated glucose uptake in tankyrase 1 knockout WAT is normal while the sensitivity of insulin-stimulated glucose uptake in tankyrase 2 knockout WAT is decreased. (A) Assay for insulin-responsive glucose uptake in TANK1^+/+^and TANK1^−/−^ WAT cells. The results shown represent three independent experiment, each using two male TANK1^+/+^ and 2 male TANK1^−/−^ mice. (B) Assay for insulin-responsive glucose uptake in TANK2^+/+^and TANK2^−/−^ WAT cells. The results shown represent five independent experiments, each using two male TANK2^+/+^ and 2 male TANK2^−/−^ mice.

Human tankyrase 1 has been reported to upregulate the translocation of Glut4 from endosomal compartments to the plasma membrane in response to insulin stimulation [15], which suggested that tankyrases might be involved in insulin-dependent glucose metabolism through their effect on GSV. To test whether mouse tankyrases play a role in insulin-dependent glucose metabolism, insulin-stimulated glucose uptake was therefore examined in WT, TANK1^−/−^ and TANK2^−/−^ WAT cells. Glucose uptake was increased comparably in TANK1^−/−^ and littermate WT control WAT cells in response to insulin stimulation (Fig. 7A). Insulin-stimulated glucose uptake in TANK2^−/−^ WAT cells tended to be reduced in comparison to its littermate control WAT cells (Fig. 7B), but this difference was not statistically significant (p = .19).

### Embryonic lethality resulted from TANK1 and TANK2 double deficiency

**Figure 8:**
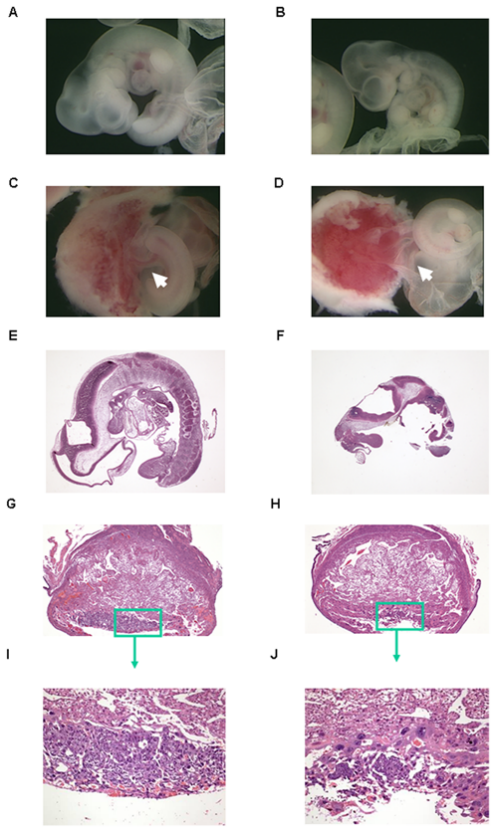
Abnormal phenotypes in TANK1^−/−^.TANK2^−/−^ mouse embryos and placentas. (A, C) wild-type and (B, D) TANK1^−/−^.TANK2^−/−^ E10.5 embryos and placentas. Arrow points to umbilical cord. Histological sections of wild-type (E) and TANK1^−/−^.TANK2^−/−^ (F) E10.5 embryos. Transverse sections of E10.5 wild-type (G) and TANK1^−/−^.TANK2^−/−^ (H) placentas. The box area in (G) and (H) are shown at a higher magnification in (I) and (J), respectively.

It remained possible from analysis of TANK1^−/−^ and TANK2^−/−^ mice that tankyrase 1 and tankyrase 2 mediate important functions that are obscured by redundancy in the functions of these molecules. To directly address this possibility, tankyrase 1 and 2 double knockout mice were generated by intercrossing TANK1^−/−^ and TANK2^−/−^ mice. No adult TANK1^−/−^TANK2^−/−^ mice were observed in 194 offspring from this intercross (Table 1), indicating that double inactivation of tankyrase 1 and 2 resulted in mouse embryonic lethality, and that tankyrase is essential for mouse development. Of interest, there were also no TANK1^+/−^TANK2^−/−^ mice in the 194 offspring, indicating that haploinsufficiency of TANK1 is lethal in the absence of TANK2. To determine the time of death during embryonic development of TANK1^−/−^TANK2^−/−^ or TANK1^+/−^ TANK2^−/−^ mice, embryos were isolated from TANK1+/−TANK2+/− intercrosses at embryonic (E) days 12.5 and 10.5, and individual embryos were genotyped by PCR. No TANK1^−/−^TANK2^−/−^ or TANK1^+/−^TANK2^−/−^ mice were identified in sixty-five E12.5 embryos (Table 1). Four TANK1^−/−^TANK2^−/−^ and four TANK1^+/−^ TANK2^−/−^ embryos were found in fifty E10.5 embryos from the same cross. Of the four E10.5 TANK1^−/−^TANK2^−/−^ embryos observed, one was completely resorbed. The other three E10.5 TANK1^−/−^TANK2^−/−^ embryos and the four E10.5 TANK1^+/−^TANK2^−/−^ embryos (Figure 8b) were pale and smaller than wild type controls (Figure 8A).

**Table 1:** Genotype of offspring from intercrosses between TANK1^+/−^ TANK2^+/−^ mice.

	Adults	Day 12	Day 10
TANK1^+/+^TANK2^+/+^	19	4	4
TANK1^+/+^TANK2^+/−^	20	6	2
TANK1^+/+^TANK2^−/−^	19	7	2
TANK1^+/−^TANK2^+/+^	39	22	10
TANK1^+/−^TANK2^+/−^	57	24	11
TANK1^+/−^TANK2^−/−^	0	0	4
TANK1^−/−^TANK2^−/−^	27	8	7
TANK1^−/−^TANK2^−/−^	13	5	6
TANK1^−/−^TANK2^−/−^	0	0	4
**Total**	**194**	**65**	**50**

Further analysis revealed that all 7 of these embryos exhibited absence of umbilical vessels (see arrow in Figure 8D) in contrast to wild-type embryos of the same age (Figure 8C). Histological sections showed that E10.5 TANK1^−/−^TANK2^−/−^ embryos were severely deteriorated in comparison with wild type controls (Figure 8E, F). Analysis also revealed abnormal placental development in TANK1^−/−^TANK2^−/−^ embryos. Figure 8 illustrates that E10.5 TANK1^−/−^TANK2^−/−^ embryos had fewer trophoblast cells in the developing placenta (Figure 8H, J) than E10.5 wild-type embryos had (Figure 8G, I). Although the cause of lethality of TANK1^−/−^TANK2^−/−^ or TANK1^+/−^TANK2^−/−^ embryos is not fully defined, the lack of umbilical cord and failure of placental development may constitute the cause of failed embryonic development.

## Discussion

Human tankyrases have been reported to be critical for telomere length maintenance, sister telomere separation, and mitotic progression, and it has been demonstrated that these functions are mediated through their poly(ADP-ribose) polymerase (PARP) activity [10], [13]. It has also been suggested that tankyrases regulate translocation of Glut4, thus playing a critical role in glucose metabolism [15], [16]. We have carried out a systematic analysis of tankyrase function in vivo by analyzing the effects of inactivating either or both tankyrase genes in mice.

The most extensively studied aspect of tankyrase function has involved the role of tankyrases in regulation of telomere length. In contrast to what has been observed in *in vitro* studies of human tankyrase function, we recently reported that neither complete deficiency of mouse tankyrase 2 nor mutation in its PARP domain affects the maintenance of telomere length *in vivo* [6], [18]. To further assess the function of mouse tankyrases, we generated tankyrase 1 knockout and tankyrase 1/2 double knockout mice. In the present study we found no defects in telomere length maintenance in tankyrase 1 deficient mice. These findings could reflect the absence of any role for tankyrase in telomere length regulation in mice, in contrast to the function of telomerase, which is functionally conserved in humans and mice. The function of human tankyrases in modulating telomere length has been linked to their ability to bind to TRF1. Mouse TRF1 lacks a tankyrase binding motif, consistent with the possibility that tankyrase function in telomere maintenance differs with species (11, 16, 20, 21), and that tankyrases do not play a role in telomere length regulation in mice. The lack of effect on telomere length in either TANK1^−/−^ or TANK2^−/−^ single knockout mice could also reflect functional redundancy rather than lack of function of these two molecules. The fact that TANK1^−/−^.TANK2^−/−^ double deficiency results in embryonic lethality in fact indicates that there is redundancy of these genes for one or more functions critical for viability, as discussed below, but complicates analysis of potentially redundant functions in telomere maintenance. Studies of conditional knockout mice and/or siRNA treatment of either TANK1^−/−^ or TANK2^−/−^ cells could provide information on the nature of redundant function.

It has been shown that tankyrase 1 is necessary for successful resolution of sister telomere association at mitosis in human cells [13], [14], and that knockdown of tankyrase 1 results in mitotic arrest [12], [13]. In contrast, in the studies reported here, we have demonstrated that inactivation of tankyrase 1 or tankyrase 2 has no effect on mitosis in the mouse cells analyzed. It was recently reported that depletion of TRF1 and TIN2 abrogates the requirement for tankyrase 1 in human sister telomere resolution and mitotic progression [14]. Thus, it is possible that our failure to observe an effect on mitosis in tankyrase-deficient mouse cells reflects the absence of interaction between mouse TRF1 and tankyrases. As noted above, an alternative explanation is the existence of redundant functions of tankyrase 1 and tankyrase 2 in sister telomere resolution and mitotic progression. Of note is the finding that embryonic lethality of TANK1/TANK2 double knockout mice does not occur until embryonic day 10. It would be anticipated that any profound defect in mitosis in these mice would be lethal at a very early stage and would prevent development to this point unless alternative tankyrase-independent mechanisms are active during early embryogenesis. There may therefore be a profound species difference in the requirement for tankyrase during mitosis.

We previously reported that tankyrase 2 deficiency results in a reproducible decrease in overall body weight [6], [18]. In contrast, there was no decrease in body weight in TANK1^−/−^ mice. Although the basis for alteration in body weight is not clear, it may be related to changes in overall metabolism, potentially including tankyrase-dependent regulation of GSV. Deficiency of mouse tankyrase 1 did not affect body weight in TANK1^−/−^ mouse in contrast to the effect seen in TANK2^−/−^ mice. Insulin-stimulated glucose uptake tended to be reduced in TANK2^−/−^ WAT cells, but this difference was not statistically significant, while TANK1^−/−^ WAT cells have normal sensitivity of insulin-stimulated glucose uptake. A comprehensive analysis of *in vitro* and *in vivo* glucose metabolism in tankyrase 2-deficient mice is currently in progress (manuscript in preparation).

The studies presented here also revealed the existence of a previously unappreciated TANK1 gene product, designated TANK1a, encoding an HPS domain-deleted TANK1 protein that was observed in mouse testis. The mRNA encoding TANK1a was transcribed by an alternative promoter of the TANK1 gene. The expression of TANK1a was tissue-specific, with mRNA and protein detected in testis, but not other tissues tested, in contrast to the apparently ubiquitous expression of tankyrase 1 and tankyrase 2 mRNA. The function of TANK1a will require further study.

Although development of TANK1^−/−^ and TANK2^−/−^ single deficient mice was generally normal, TANK1^−/−^TANK2^−/−^ and TANK1^+/−^ TANK2^−/−^ genotypes were embryonic lethal at E10.5 (Table 1), and the TANK1^−/−^ TANK2^+/−^ genotype was partially lethal (Table 1)These findings indicate that tankyrase 1 and 2 mediate redundant functions that are critical to embryonic mouse development. Although the underlying mechanism of this lethality is unclear, histological study indicated that TANK1^−/−^TANK2^−/−^ and TANK1^+/−^ TANK2^−/−^ embryos lacked umbilical cords and that placental development was abnormal, factors which are sufficient to result in the death of embryos.
